# Action of Mechanical Forces on Polymerization and Polymers

**DOI:** 10.3390/polym14030604

**Published:** 2022-02-03

**Authors:** Anatoly T. Ponomarenko, Alexey R. Tameev, Vitaliy G. Shevchenko

**Affiliations:** 1 Enikolopov Institute of Synthetic Polymeric Materials, Russian Academy of Sciences, 70 Profsoyuznaya, 117393 Moscow, Russia; anapon@ispm.ru (A.T.P.); shev@ispm.ru (V.G.S.); 2 Frumkin Institute of Physical Chemistry and Electrochemistry, Russian Academy of Sciences, 31, bld. 4 Leninsky Prospect, 119071 Moscow, Russia

**Keywords:** mechanochemistry, polymerization, radicals, anvils, mechanodestruction, mechanophores, rheological explosion, radiofrequency superradiance

## Abstract

In this review, we summarize recent developments in the field of the mechanochemistry of polymers. The aim of the review is to consider the consequences of mechanical forces and actions on polymers and polymer synthesis. First, we review classical works on chemical reactions and polymerization processes under strong shear deformations. Then, we analyze two emerging directions of research in mechanochemistry—the role of mechanophores and, for the first time, new physical phenomena, accompanying external impulse mechanical actions on polymers. Mechanophores have been recently proposed as sensors of fatigue and cracks in polymers and composites. The effects of the high-pressure pulsed loading of polymers and composites include the Dzyaloshinskii–Moriya effect, emission of superradiation and the formation of metal nanoparticles. These effects provide deeper insight into the mechanism of chemical reactions under shear deformations and pave the way for further research in the interests of modern technologies.

## 1. Introduction

In recent years, there has been a dramatic increase in research into the mechanochemistry of polymers. In the past three to four years, several excellent reviews have been published on this topic. Deneke et al. [1] highlighted recent progress in mechanophores (MPs) in polymeric systems and their potential applications as stress sensors due to a color change and/or fluorescence activation under an applied load. Creton and Sijbesma et al. [2] exhaustively summarized fruitful synthetic methods for incorporating various mechanosensitive molecules into polymer structures, as well as application areas of such polymers. The existing toolbox of MPs and related theoretical methods were presented in the comprehensive review by Ghanem et al. [3]. Changes in the kinetics of polymerization, monomer selectivity and polymer tacticity modulated by external stimuli including mechanical force were highlighted by Doerr et al. [4]. A hierarchy of mechanochemical phenomena, which may guide the development of multiscale models of mechanochemical reactivity to match the breadth of the Eyring equation of chemical kinetics, was proposed by Akbulatov and Boulatov [5]. Izak-Nau et al. considered polymer mechanochemical pericyclic reactions [6]. H. Hu et al. [7] presented a systematic review for polymer mechanochemistry-based reaction cascades, which involves at least two subsequent chemical reactions, with the first one induced by mechanical force. Atomic force microscopy (AFM) is widely used in mechanochemistry studies. Liu and Vancso [8] reported on studies on the nanoscale by AFM that are intrinsically related to covalent macromolecular chains, their direct observations, molecular forces, and processes. De Bo [9] focused on the mechanochemical investigation of interlocked structures by AFM and polymer mechanochemistry (under ultrasound). Recent advances in the ultrasound-mediated (the atom transfer radical polymerization) ATRP were discussed by Zaborniak and Chmielarz [10]. The review by Zhou et al. [11] presented a comprehensive analysis on the polymerization systems under various physical modulations, including the consideration mechanoATRP under ultrasound agitation.

In this review, we consider basic knowledge and recent developments in the mechanochemistry of polymers and, unlike previously published reviews, we treat classical results from the contemporary point of view and, for the first time, summarize new developments in pulsed mechanochemistry.

Of the classic problems, we discuss the consequences of mechanical actions on polymers and polymer synthesis occurring on Bridgman anvils, in extruders and mills, as well as effects of ultrasound and crazing phenomena. Investigated materials were in different aggregation states. For analysis phenomena in solid polymers subjected to mechanical stress, appropriate approaches have been developed in the chemical physics of solids. In liquid media, a specific physical process, cavitation, takes place, which, due to its high-energy intensity, causes many different consequences, as shown in the review. We pay some attention to the crazing phenomenon as a method associated with the mechanical deformation of polymer-inducing nanoscale porosity. Porosity develops due to the interaction of the formed porous structure with an adsorption-active medium (active liquid). Crazing can essentially be attributed to a mixed type of action on polymers, including “external” mechanical action and then “internal” interchain action on the nanoscale. The latter is also used to create polymer mixtures incorporating highly dispersed small molecules. The classic issues are considered in the following order: the main basic areas of mechanochemistry (Section 2), including the relationship between chemistry and technology (Section 3), current approaches to the implementation of mechanochemical effects (Section 4), and the mechanism of mechanochemical reactions in initial stages (Section 5).

Of the emerging trends, two new areas of mechanochemistry are analyzed in Section 6: the role of mechanophores, which we treat from the point of view of chemical transfor-mations under static mechanical loading, and, for the first time, new developments in pulsed mechanochemistry including physical phenomena that accompany the pulsed mechanical action on polymers. Mechanophores have been recently proposed as sensors of fatigue and cracks in polymers and composites. The effects of the high-pressure pulsed loading of polymers and composites include the Dzyaloshinskii–Moriya effect, emission of superradiation and the formation of metal nanoparticles. These effects provide deeper insight into the mechanism of chemical reactions under shear deformations and pave the way for further research in the interests of modern technologies.

## 2. The Main Directions of Research in the Mechanochemistry of Polymers and Their Current State

Previously, the scientific literature summarized mainly works related to such an important section of mechanochemistry as the effect of ultrasound on various objects, including polymers. These include papers in the book edited by Rudi van Eldik and Colin D.Hubbard [12,13,14] and paragraphs in the reviews [10,11]. There is important information given in the review by Ponomarenko et al. [15] on methods for making fibers by essentially mechanochemical methods, such as electro- and magnetospinning, respectively, in electric and magnetic fields, although in the latter case there was no mention of mechanochemistry. The monographs by Balaz [16] and review by Caruso et al. [17] are also devoted to the consideration of mechanochemistry issues. This review [17] covers many aspects and details of polymer mechanochemistry; it is the most complete of the currently published reviews, yet it misses some publications that have made a significant contribution to the theory and practice of polymer mechanochemistry. It is worth paying attention to the first monograph by Khait et al. on the topic [18], in which the original research carried out by Enikolopov and his collaborators is reviewed with extreme details. In technology developments, this team also obtained important results in the field under consideration [19].

It follows from many studies that, since the reactivity of solids is limited by the low mobility of the elements they consist of, most of the chemical technological processes used include liquid and gaseous phases. However, the high mobility of these phases creates problems: it is difficult to maintain tightness of the reactors, isolate reaction products, and store waste. In this regard, chemical processes involving solids have advantages and mechanical action on solids in many cases is a convenient way to ensure the mobility of solid body elements at the time of transformation and to store the energy necessary for chemical transformations [13,14,15,16,17,18]. The use of mechanochemistry is also important for solving environmental problems, being based on the use of various types of mechanochemical effects. Mechanical action on solids leads both to their dispersion and to the formation of defects in the crystal lattice. Powders of solids with a larger surface react faster both with dissolved substances and with each other. The accumulation of defects, as well as active elements in the form of radicals, usually increases the reactivity of solids. Pretreatment of solids also accelerates their interaction with gases and liquids. The use of the interaction of solids with each other under mechanical action makes it possible in some cases to exclude water and other solvents from the technology and, respectively, to exclude the discharge of liquid waste into the environment [16,17]. The review presents some examples of the implementation of this approach, namely, in the chemical industry. Apparently, the development of work in this area will allow us to get closer to the most effective way of eliminating environmental problems by creating waste-free and low-waste technologies.

Mechanical action on mixtures of solids and liquids dramatically accelerates their interaction. There are indications that conditions close to hydrothermal conditions can be realized in mechanical shock activators during the processing of building mixes. Processes based on these effects are also used in the chemical, pulp and paper industries and industrial recycling of various wastes, including polymers [16].

The analysis shows that mechanochemistry is a very important area of research, and, to date, extensive information has been accumulated in terms of both volume and the results of its use in scientific and practical aspects. These works are very important during the transition from synthesis in a laboratory flask, on a small scale, as well as during its implementation in the laboratory and further in industrial reactors, as well as during the transition to the mass production of certain products made of polymers and polymer composite materials. This judgment is clearly seen in many of the abovementioned reviews. The research is developing in the following directions:

(1) Study of the mechanisms and energy of chemical transformations in various mechanical fields.
(2) The use of mechanochemical reactions for the synthesis and modification of polymer materials.
(3) Search for ways to inhibit undesirable mechanochemical side processes that cause a drop in strength, increased wear and premature failure of various parts and structures made of polymer materials during their operation in static and dynamic mechanical fields.

Some technical solutions, after solving the issues of scaling and selecting appropriate high-performance devices, are applied in practice. For example, thermomechanical processes of lignin extraction, until recently used in the paper industry, with some modifications, are used to separate lignin from cellulose and increase the rate of subsequent enzymatic hydrolysis, obtaining bioethanol and animal feed components. 

However, the cost-effective processing and recycling of polymers and plant materials is complicated by the lack of basic knowledge about heterogeneous transformations, the structure of polymers, and the multilevel composition of plant materials. The latter have not only disordered and reactive, but also ordered fragments that need activation as shown in the reviews by Boldyrev [20] and Boldyreva [21]. Mechanical methods look attractive in terms of the environmental impact, the availability of industrial equipment on the market, and the ease of operations to increase the reactivity of natural polymers. Mechanochemical technologies generally do not use liquid-phase methods (dissolution, recrystallization, leaching, and evaporation). They make it possible to manufacture products in a solid powder state, which greatly simplifies the processing of raw materials, storage and transportation of finished products [17].

The increase in reactivity by mechanical activation is carried out due to the so-called active states of the solid. Currently, it is believed that the relaxation of the field of mechanical stresses, and hence the energy transferred to the material, proceeds mainly due to grinding (formation of a new surface), elastic and plastic deformation, the formation of defects in the crystal structure, local growth of pressure and temperature, acceleration of mass transfer processes. In some cases, mechanical loading leads to the formation of metastable polymorphic modifications of substances that persist even after the cessation of mechanical action [20,21,22].

From the standpoint of solid-state chemistry, the main factors responsible for reactivity in heterogeneous reactions are chemical composition and the properties of the newly formed surface, which is the boundary of the solid–liquid interface. Thus, the study of the processes occurring during the mechanical processing of polymers of natural origin is also an urgent and in-demand task from the point of view of modern science and technology. The knowledge gained in the field of changing the structure and properties of natural polymers is in demand in solid-state chemistry and is necessary for solving applied problems of modern chemical technology.

The purpose of many research works was to study the physico-chemical processes occurring during the mechanochemical processing of solid-state polymers, polymeric supramolecular complexes and materials. The purpose of applied research was the development of technologies for the mechanochemical processing of natural polymer raw materials into products in demand on the market. To achieve these goals, the following important tasks are under implementation:

1. Study of physico-chemical processes occurring in natural polymers, their supramolecular complexes and biological materials during mechanochemical processing in laboratory and industrial conditions [23];
2. Obtaining information about the influence of the chemical composition, surface properties, crystal and supramolecular structures of carbohydrate, protein and polyphenolic polymers on their reactivity in heterogeneous reactions, which follow mechanochemical processing [24];
3. Finding the conditions of mechanochemical processing of protein macromolecules, to ensure the stability of proteins and leading to the formation of reactive composites with carbohydrate polymers and vegetable raw materials in general [25];
4. Investigation into the behavior of nanocomposites and micro-composites based on polystyrene under conditions of rapid compression and identification of the role of mechanical action in the combined thermomechanical process [26];
5. Carrying out the mathematical modeling of vibration effects on the kinetics of solid-phase decomposition reactions in condensed media to develop models of the mechanism and reveal the role of mechanical action in the combined mechanochemical process [27];
6. Propose a mechanism revealing the role of mechanical action in the overall mechanochemical process, including mechanochromism [1,28,29,30,31,32];
7. Systematization of macroscopic manifestations of polymer mechanochemistry and evaluation of results in the framework of physical chemistry [5].

The theoretical significance of the above works lies in the expansion of fundamental knowledge about the nature of physico-chemical processes occurring in natural polymers (proteins, carbohydrates and polyphenols), their supramolecular complexes and real materials during mechanochemical processing in laboratory, semi-industrial and industrial conditions. The final task is to implement at the semi-industrial and industrial level methods of mechanochemical processing of natural and synthetic polymer raw materials into demanded products: biofuel components, synthetic antibiotic substitutes, and complex heavy metal sorbents.

In the technology of processing polymers into products, a number of techniques have been developed to increase the speed of their molding. This technique is called polymer plasticization—thanks to which their further processing is facilitated. There are two known methods of plasticization—mechanical and thermo-oxidative—and the latter is carried out without mechanical action. In the reviews by Prut and Zelenetskii [33] and Fridman and Prut [34], it is believed that mechanical plasticization is more often used in industry, resulting in a change in the functional composition and a decrease in the molecular weight of polymers. Other changes in the same direction include oxidation of the end groups, changes in the strength of polymers, for example, elongation and tear resistance and elastic modulus, due to a decrease in molecular weight, and the value of each of these parameters is closely related to the chemical structure of a high molecular weight compound. It should be kept in mind that the structural side of polymer mechanochemistry is based on the ideas of the connection of these effects with the loss of stability during their deformation, as Peterson et al. [35] and Aleksandrov et al. [36] considered.

Along with the directed mechanical actions discussed above, mixing is used in technological practice as developed by Willis-Fox et al. [37]. Extensive and very detailed information is contained in one of the first monographs on this topic by Khait et al. [18], which highlights many important aspects of such new methods of polymer processing as their solid-phase grinding. 

An example of modern solid-phase synthesis in a twin-screw extruder is the production of a biocompatible nanocomposite for tissue engineering based on allylchitosan and vinyltriethoxysilane studied by Aleksandrov et al. [36]. The research led to technology that has been developed, and many details of the mechanism of the synthesis of a biocompatible nanocomposite have been established, which is promising for use in regenerative medicine and tissue engineering.

## 3. Chemical and Technological Aspects of Mechanochemical Transformations of Polymers

The real chemical and technological aspects of polymer mechanochemistry are covered in a number of reviews. A distinctive feature of the reviews by Prut and Zelenetskii [33] and Fridman and Prut [34] is that despite their publication many years ago, the relevance of the information has not been lost to this day. So, chemical problems of polymer mechanochemistry are still relevant today, such as the processes of chemical modification and mixing of polymers occurring in extruder-reactors, as well as the analysis of the relationships between the mixing time, chemical reaction and residence of the reaction mixture in the extruder-reactor, which affect the kinetics of reactions and the structure of the resulting products. The review [34] also considers the principles and processes of processing polymers and composite materials, taking into account the directed effects of mechanical fields on the rheological properties of polymers. At the same time, it is shown that this is a promising way to create new molding processes for products made of polymers and composite materials. Techniques such as solid-phase extrusion and hydroextrusion, cyclic shear deformation of melts, as well as the effects of shear and ultrasonic vibrations are important. This way allows for obtaining heavy-duty films and fibers. When forming products, friction at the polymer–mold interface plays a very important role, and to reduce the coefficient of friction, it is effective to bring ultrasonic low-amplitude vibrations with a frequency of about 20 kHz to one of the friction pairs, which reduces the coefficient of friction to almost zero, thereby providing a significant intensification of solid-phase extrusion. An important role in the creation of new products is played by such a technique expressed in the review as the method of two-layer solid-phase co-extrusion, which subsequently allows for intensifying the process of molding products and reducing overall energy consumption despite the additional generation of ultrasonic vibrations, especially when processing highly filled thermoplastics. As the authors note, this technique proved to be particularly effective in the processing of asbestos-filled thermoplastics. The effect of shear ultrasonic vibrations on polymer melts is relatively simple to create with the help of rod- or disc-shaped emitters mounted on extrusion heads. The efficiency of this method of forming thin sleeve films up to 10 microns thick, which is now widely used, turned out to be very high.

**Figure 1:**
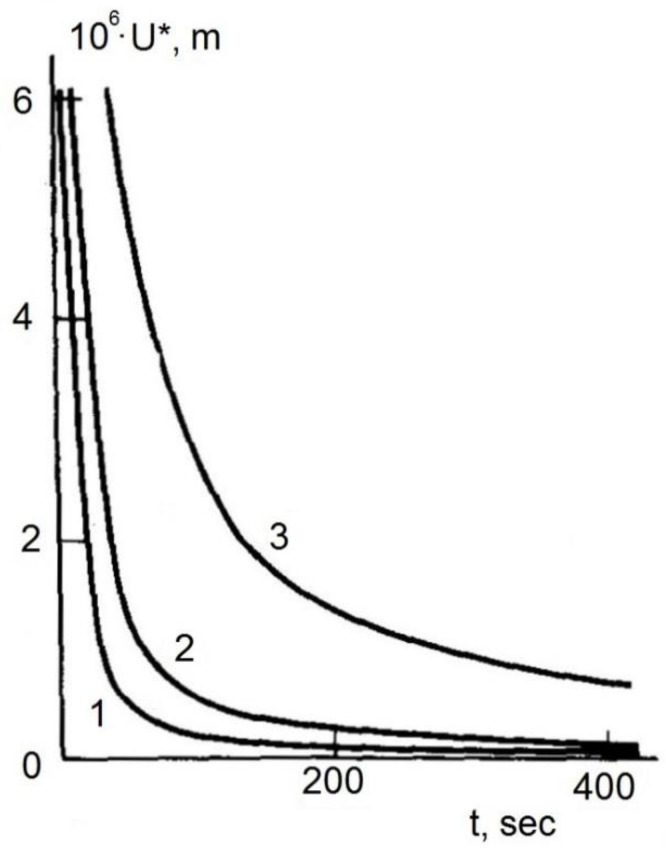
Amplitude of acoustic waves U* vs. treatment time *t* for monodisperse polybutadienes of molecular weight (MW) 1.6 × 10^5^ (1); 3.5 × 10^5^ (2) and 5.3 × 10^5^ (3) [34].

Considering the role of acoustic cavitation, the main work in this direction was carried out in the period 1978–1983, and their most important result was the conclusion that it was necessary to exceed the acoustic pressure over the volumetric strength of the polymer and that for this purpose it is necessary to use volumetric ultrasonic vibration. The phenomenon of acoustic cavitation of polymers and composites is especially important in high-viscosity materials processing technologies, since with increasing viscosity, the average size, growth rate and collapse of cavitation cavities decreases, and therefore the potent effects of cavitation are suppressed. Thus, when the viscosity of the mixture is above 1.5–2 Pa·s, the secondary effects of acoustic cavitation cease to be observed. According to Fridman and Prut [34], acoustic cavitation has a threshold character, i.e., first, for each polymer, there is a critical amplitude of acoustic vibrations U*. These dependences of U* on *t* are satisfactorily described by a power function at small amplitudes of volumetric ultrasonic vibrations and an exponential function at relatively large amplitudes (Figure 1).

**Figure 2:**
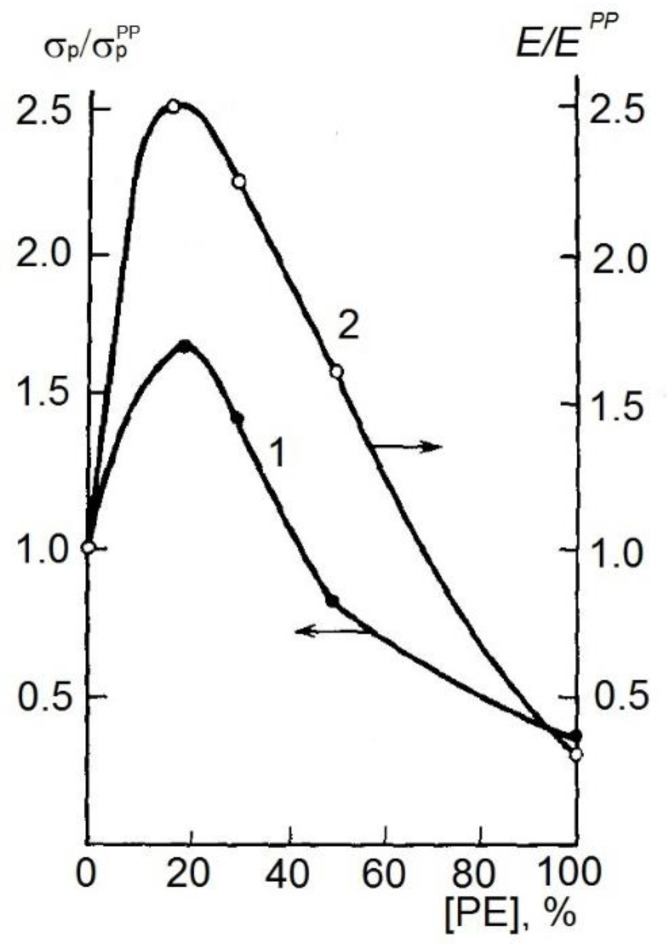
Dependences of the tensile strength σ_p_ (1) and the elastic modulus *E* (2) on the content of PE in the composition of the polypropylene—high-density PE composition at a stretch ratio of 15 and temperature of 120–130 °C. σ_p_ and *E* normalized to those for polypropylene σ_p_^PP^ and *E*^PP^, respectively [34].

The excitation of cavitation, secondly, in melts of industrial polymers leads to a sharp decrease in the effective viscosity of thermoplastics melts and highly filled composites, and depending on the nature of the polymer, the viscosity coefficient of the polymer can decrease by 5–100 times. It is important that in this case there is an irreversible increase in the effective fluidity of the material processed in the acoustic field. Thirdly, in addition to these effects, there are noticeable changes in the structure of the resulting extrudates, such as the average size of structural elements, their size distribution narrows, and in the case of processing composites, the uniformity of particle distribution and their grinding increases at a certain brittleness. The section of the review that discusses methods for obtaining heavy-duty, high-modulus and ultrathin films and fibers deserves attention and appreciation, since these forms of polymers and products made from them are of crucial importance in polymer materials science. This can be achieved by straightening polymer chains and, in this case, the value of the elastic modulus may correspond to the theoretically calculated value; however, there may be inconsistencies due to some structural defects. Fridman and Prut [34] rightly note that a positive result is achievable with the use of multi-stage orientation extraction when optimizing the polymer structure in the initial material, as well as the method of orientation crystallization, and the use of thermoplastics with a liquid crystal structure. These ideas found their embodiment back in the 1980s when Enikolopov and coauthors prepared films of polypropylene with a thickness of 30–40 microns, with a tensile modulus Δ*L* of 1.3 to 1.5 GPa and a tensile strength of up to 36–43 GPa [38]. So, high values of the elastic modulus, which turned out to be three times greater than steel, the authors quite reasonably believe, were achieved due to a large degree of orientation of polymer chains, a change in the structure of the polymer in amorphous regions, and an increase in the number of transient chains in such materials. Polymer–polymer composite materials, such as polypropylene–polyethylene mixtures, can serve as another class of polymer materials promising for the production of high-modulus films. From the data shown in Figure 2, it follows that the use of polymer–polymer mixtures makes it possible to obtain materials with higher physical and mechanical properties.

Attention should be paid to the concepts of the electrodynamic and thermo-mechanical instability of polymers, leading to the self-organization of polymer systems according to Volynskii and Bakeev [39]. Self-organization in amorphous polymer systems is a universal phenomenon and can occur under the action of external forces of a different nature, such as, for example, electric fields, as shown by Schäffer et al. [40]. This paper reports that a thin layer (100–200 nm) of amorphous polymer is deposited on a silicone substrate with a flat electrode serving as one of the capacitor plates, and located at a distance of 100–1000 nm from the first. Then, the entire device is heated to the melting temperature of the polymer, for example, up to 170 °C for polystyrene, and a voltage of about 20–50 V is applied to the plates of such a capacitor, with an electric field strength of 10^7^–10^8^ V/m and a current density of 10–50 mA/cm^2^. The mechanism of instability of the system under consideration, proposed by Volynskii and Bakeev [39] and Schäffer et al. [40], consists in the fact that the polymer film is subjected to competition between the actions of mechanical tension and electric field and the period of identity may vary depending on their ratio. From the consideration of the above phenomena, it follows that the loss of stability under the influence of an external field is a precursor of deeper changes in the object, for example, as a probable precursor of an electrical breakdown. At the same time, an amazing strictly regular structure is observed, and after cooling below the glass transition temperature, it can be investigated using direct microscopic experiments. The authors believe that the mechanism of instability of the system consists of a critical balance of forces at the polymer–air interface due to the fact that the polymer film is subjected to surface tension, which minimizes the interface area, and, on the other hand, by an electric field that polarizes the dielectric. In this regard, atomic force microscopy, a well-known powerful tool for surface imaging, is extremely useful for the basic research of mechanical [41,42,43,44,45,46,47] and electrical [48,49,50] heterogeneities in polymers and related matter.

## 4. Actual Sources and Means for the Implementation of Mechanochemical Effects

First, it should be noted that mechanical effects on substances can be divided by the nature of mechanical forces into two large groups—shear and shock—with many of their varieties [5,51,52]. A number of techniques and devices have been developed for the implementation of mechanochemical reactions, such as Bridgman anvils [53,54,55], ball mills [56], bead mills, hammer mills, disc mills, jet mills, extruders of various types. It is essential that in each such device, the energy levels for conducting a mechanochemical reaction differ significantly. Consideration of the variants of these effects on Bridgman anvils, in particular polymerization reactions, is of particular interest due to the extremely unusual kinetics of the process [55]. Based on numerous studies and analysis of the data presented in the reviews by Enikolopov [53] and Zharov [55], the following conclusions are made:

(1) The rate of chemical transformation in solids during shear deformation is very high and 3–8 orders of magnitude higher than the rate in the liquid phase;
(2) There is no need to use a catalyst for the reaction to proceed;
(3) The yield of the chemical reaction is proportional only to the magnitude of the shear strain, and the rate of the chemical reaction is proportional to the rate of deformation;
(4) It turned out that there is no reason to use the generally accepted concept of the diffusion coefficient to quantify the mass transfer rate. If we use the “concept of reduced diffusion coefficient”, then we can make sure that its value at the moment of shear is 8–10 orders of magnitude higher than without shear deformation and 3–5 orders of magnitude greater than the diffusion coefficient of the same molecules at the same temperature in the liquid phase.

Thanks to the use of MPs, it has been established by Black et al. [57] and Lenhardt [58] that any mechanical action leads to the occurrence of chemical transformations in the object of mechanical action.

A separate and very productive method of mechanochemistry is the so-called wave effects on polymers and composites, which in some cases are used along with the above effects and are associated with the use of ultrasonic effects. The active effect on the polymer, leading to irreversible changes in its composition and structure, is due to nonlinear effects in an ultrasonic field [59]. Ultrasonic impacts are now very widespread and a search is underway to expand their application in modern technologies. In particular, probing the mechanophore’s activity by ultrasonication and/or by nanoscale single-molecule force spectroscopy (SMFS) experiments provides a way to optimize solid-state activation methods for polymers as shown by Deneke et al. [1], Radiom et al. [60,61] and Martínez-Tong et al. [62]. Mechanochemical transformations under the action of ultrasound and SMFS are clearly manifested when using multi-MP polymers, as shown by Bowser and Craig [63]. A polybutadiene sample functionalized with gem-dibromocyclopropane mechanoactive segments with the goal of obtaining a system with stress-relief capabilities was studied by Wu et al. [64] using SMFS and the functionalized polymer chains were found to present a 28% additional strain, allowing the systems to remodel under tension. These SMFS results opened the possibility of studying the mechanical behavior of other cyclopropane-based systems as shown by the Craig group [65,66].

In [67], Enikolopov et al. indicated that solid-phase chemical reactions proceed at a higher rate if the reaction mixtures are preheated to very high temperatures (this is how piezoceramics, high-temperature superconductors, etc., are synthesized). On the other hand, this technology is very energy-intensive, the reaction rate is low, and, therefore, the authors searched for a positive result by using ultrasonic action in two-, three-, and four-component mixtures, which in the liquid-phase version can be carried out only in several stages. Enikolopov et al. [67] reported a successful attempt to synthesize an azo dye from aniline hydrochloric acid and sodium nitrate in the form of powders with a particle size of 50–100 μm. It was found that within 2–3 s from the start of sonication with an amplitude of 10 μm the tablet acquired a yellow color and after 200 s the reaction was completed at 100% conversion. No less surprising are the results of the ultrasonic stimulation of a solid-phase synthesis reaction from a compressed four-component mixture of beta-naphthol, n-nitroaniline, potassium hyposulfite and potassium nitrate powders. This reaction was completed in 360 s with an oscillation amplitude of 15 μm. Thus, Enikolopov et al. [67] convincingly showed that decomposition, substitution, and synthesis reactions can proceed at a certain vibration amplitude (characteristic of a particular reaction), provided that the initial reagents are in the form of tablets. In this case, the reaction rates are close to those observed under shear pressure, and several orders of magnitude higher than the rates in the liquid phase. The authors believe that diffusion restrictions are reduced by ultrasound. In our opinion, the reality of such a judgment requires additional experimental evidence.

The mechanochemical processes, as already reported above, can also include the established interaction of polymers with liquids, leading to crazing, which is referred to as a universal type of self-organization of amorphous polymers [39]. This phenomenon can be realized in polymer systems under the influence of a wide variety of external forces, including an electric field. Thus, it can be assumed that such self-organization precedes the electrical breakdown of thin films.

## 5. Current Concepts of the Mechanism of the Initial Stages of Mechanochemical Reactions and Their Development

The variety of types of reactions and conditions of their course and the difficulties associated with the inability to directly observe their course at the microscopic or molecular levels lead to the appearance of diverse models, each having a limited scope of applicability. Many authors believe that “the central point of mechanochemistry, like thermochemistry, is to determine the transition state of a reaction as the apex of a potential barrier that is on the path of a chemical reaction” [68]. 

**Figure 3:**
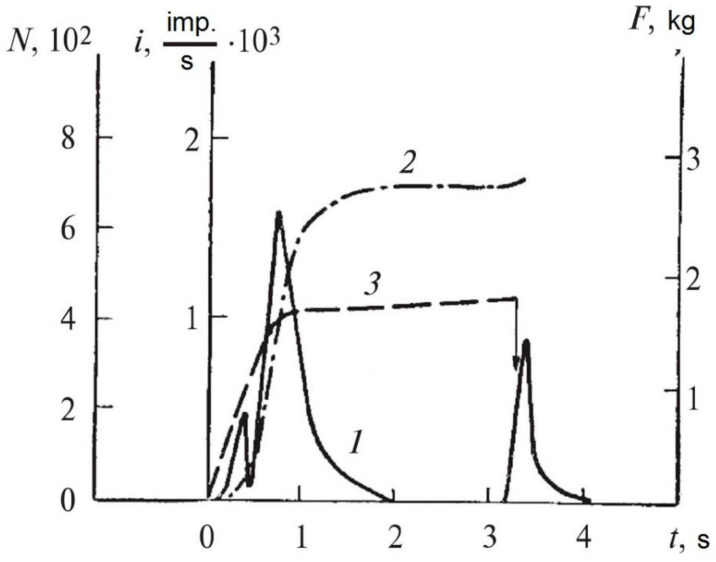
Dependence of the intensity of electron emission *i* (*1*), the total number of pulses *N* (*2*) and the load on the sample *F* (*3*) on the deformation time of 200 μm high-density PE film at 300 K and the loading rate 9.84 mm/s [68].

The above approach, both in our opinion and Kalnin’sh et al.’s [68], is evaluated as a fundamentally new way of explaining the mechanisms of synthesis and destruction of polymers under mechanical influences. The central point in mechanochemistry, as in thermochemistry, is to determine the transition state of a reaction as the apex of a potential barrier that is on the path of a chemical reaction. The reason for the electronic excitation in this case is the mechanical action initiated by the mechanical stresses created by the corresponding elements in various devices. At the same time, the experimental data can be explained using the thermofluctuation approach, according to which thermal fluctuations appear, which means that interatomic bonds are stretched to the maximum breaking elongation, due to which they can cause rearrangements of atoms and thereby lead to breaking elongation. Figure 3 shows the dependence of the intensity of electron emission under mechanical stress in vacuum, from which it follows that this dependence has a maximum, and according to curve 1, the number of emitted electrons increases rapidly, then slows down and grows rapidly again before the sample breaks. There are various explanations for this unusual phenomenon, including electrical discharges occurring between surfaces in microcracks and ionization of polymer molecules. Kalnin’sh and Panarin [68] believe that the main cause is mechanochemical electronic excitation.

The thermal fluctuation approach to explaining the strength and deformability of solids was accepted and further developed by Zarkhin et al. [69] after the establishment of a universal empirical dependence linking the magnitude of the breaking stress with the temperature *T* and time *τ_f_* before the fracture of the sample. This dependence obeys Equation (1):

(1) $$\tau_{f}=\tau_{0}\exp\frac{U_{0}-\gamma \sigma_{f}}{kT}$$

where *τ*_0_ = 10^−12^–10^−14^ s, *U*_0_ and *γ* are the activation energy and activation volume of destruction, and *k* is the Boltzmann constant. The authors also found that the stationary creep rate $\dot{\varepsilon }$ is related to temperature and stress *σ* by an equation similar to (1), but the exponent contains a minus sign:

(2) $${\dot{\varepsilon }}_{c}={\dot{\varepsilon }}_{0}\exp(-\frac{U_{0}-\gamma \sigma }{kT})$$

According to Zarkhin et al. [69], currently, studies of the mechanical destruction of polymers can be divided into two groups. The first group includes the results indicating significant differences in the products of the thermo- and mechanochemical destruction of polymers (Table 1). In the composition of the products determined by mass spectrometry, there are no heavy polymer fragments characteristic of thermal degradation. The second group, which does not fit into the thermal fluctuation concept due to the detection of high-energy molecular products of mechanical destruction of polymethylmethacrylate and cured epoxy resin, such as methyl methacrylate, acetaldehyde and occluded water, has extremely high values of kinetic energy from 0.25 to 0.7 eV, which corresponds to a temperature of 2000 to 5400 K. This result motivated the authors to carry out mechanical destruction modeling using the molecular dynamics method, which allows us to study the dynamics of various processes in the time range 10^−14^–10^−11^ s.

The main results of the study of mechanodestruction in the framework of a one-dimensional model of a polymer chain in terms of the theory of nonlinear waves are solitary nonlinear waves of the soliton type, which are the only high-energy agents of this process. This result led the authors to the conclusion that after the rupture of the stretched chain, no thermal fluctuations occur and high-energy molecular products are formed at the initial stage of destruction of the polymer chain in the time interval of 0.01−1 ps after its first rupture. Analyzing and comparing the results with experimental data, Shlenskii [27] pointed out the need to take into account the possible conversion of a part of the elastic energy stored during the stretching of the macromolecule into electronic excitation energy under significant deformations of the macromolecule both at the stage of its stretching and after rupture at the stage of intense molecular dynamics. In accordance with Zarkhin et al. [69], we point out that the experimental results and computer simulation of mechanical destruction indicate that there may be a specific mechanism of solid-phase reactions associated with the cooperative movement of atoms. As proposed according to [69,70,71], the energetics of the process of mechanical destruction of polymer chains can be represented as Equation (3) as proposed by Zarkhin et al. [69], Kats et al. [70] and Manevich et al. [71]:

(3) $$U=nD+\overset{n-1}{\underset{i=2}{\sum }}E_{i}+E_{1}+E_{n}$$

Here, *U* is elastic (potential) energy stored in the initial polymer chain at the time of the first rupture, *n* is the number of ruptures, *D* is the dissociation energy of the C-C bond, *E_i_* is the total energy of the *i*-th molecular product (*E_i_* = *E_vib_* + *E_kin_*), and *E*_1_ and *E_n_* are total energies of two remaining chain ends. As a result of the analysis of experimental data and computer modeling in these works, it is concluded that under certain conditions for some solid-phase reactions there may be a new, cooperative mechanism of elementary act, which differs from the local transition associated with a single overcoming of the energy barrier height due to thermal fluctuations. This mechanism, according to the authors, is associated with the non-activation motion of localized elementary excitation of the crystal lattice, a topological soliton describing the transition of “molecules” from one stationary state to an intermediate stationary state close to the reaction products. In this case, for exothermic reactions, a supersonic reaction rate can be observed. This model opens up new possibilities for describing solid-phase processes and allows for a qualitative description of some anomalies in solid-state chemistry.

In the review, Radtsig [72] analyzes the original results of the study of free radical processes occurring during the machining of polymers important in practical terms, such as polyethylene, polydeuteroethylene, polypropylene, polybutene-1, polyisobutylene, poly-4-methylpentene, polystyrene, and polytetrafluoroethylene. The ESR method makes it possible to monitor the development of important parameters of mechanochemical processes, to register the structure of paramagnetic centers, their spatial distribution, and to control their chemical transformations and concentration [72,73]. The corresponding parameters, the initiation rate *W*_0_ and the maximum concentration P* for some polymers are given in Table 2. It is concluded that the limiting concentration of free radicals is determined by the equality of the rates of their formation and termination, and the termination of macroradicals is due to mobility caused by plastic deformation of the polymer. This review does not describe in detail the technique of the mechanochemical experiment; it is only indicated that a vibrating mill was used with frequency in the range 30–100 Hz, thereby confirming that the polymer objects under study are “soft” matter. In particular, it has been shown that such low intensity effects, from tenths to several watts per gram of polymer, already lead to a significant accumulation of radicals at 77 K, which are the initiators of bond breaking, the strength of which is about 340 kJ per mole [72].

In recent years, reports have appeared on in situ studies of the processes of structure formation during the deformation of materials on diamond anvils, which allows one, due to the transparency of diamond, to conduct studies of the structure and properties of substances directly under ultrahigh pressures, since such information is important for a deeper understanding of mechanochemical processes.

It is reported that an SDDAC (structure-deformation diamond anvil cell)-type camera has been developed, which is designed mainly for the implementation of volumetric and surface-intensive plastic deformations (SPDs) of micro-samples with the possibility of continuous monitoring of the stress–strain state by the nature of the change in double refraction in a polarization microscope. It is confirmed that the developed SDDAC design is multifunctional and allows for X-ray structural studies of the contact–friction interaction of substances as well as the study of the composition of products during the SPD. The emergence of such new research methods means that prospects are opening up for obtaining new information about the mechanochemistry of polymers and other objects of scientific and technical interest [74].

## 6. Current Topical Trends in Mechanochemistry of Polymers

### 6.1. Mechanophores as Indicators of Mechanochemical Transformations of Polymers

Interest in modern polymer mechanochemistry has increased rapidly in recent decades, in particular due to the widespread use of MPs, which being introduced into polymers provide the possibility of acquiring information about the consequences of physical effects on polymers by pressure and tension [1,5,75,76].

**Figure 4:**
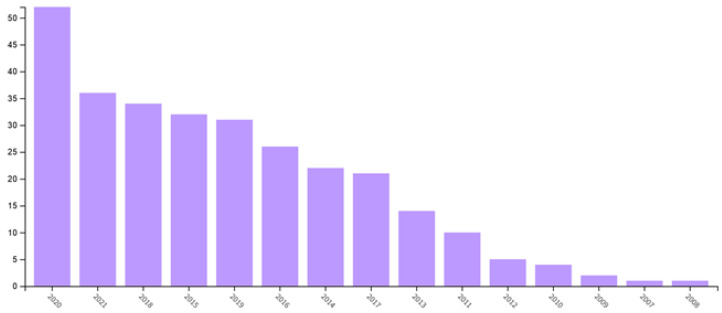
The number of papers on mechanophores (from www.webofscience.com as of 1 November 2021).

It should be noted that this direction of research belongs to the latest studies and, according to the literature, there has been a gradual increase in the number of publications in reputable journals on this topic. So, while in 2009 there were about five publications, by 2020 this figure has increased to more than 50. This can be clearly seen from Figure 4, showing the rapid growth in the number of publications from 2011 to 2013.

As Akbulatov and Boulatov [5] point out, empirical mechanochemistry has achieved great success thanks to the use of MP, but nevertheless the quantitative understanding of mechanochemical phenomena is preliminary and a lot of effort is needed to develop a concept for the rational use and systematization of the results of mechanochemical studies already found. The importance of work in this area is evidenced by the introduction of MP and barrier coatings, then it becomes possible to obtain information in advance about the critical situation with the stability of important structures made of polymer materials and composites, such as, for example, the blades of wind electric generators, components or devices of aerospace vehicles [8,76]. In this section of the review, our task is to further show the importance of work in this area and thereby stimulating interest in it by giving examples of the successful application of MP. The attractive aspects of the topics related to the engineering application of MP are described by Deneke et al. [1]. The authors note that observing the change in color or fluorescence under mechanical load allows for monitoring their operational suitability, and it is important to determine subcritical loads at which the beginning of destruction is possible in real time.

**Figure 5:**
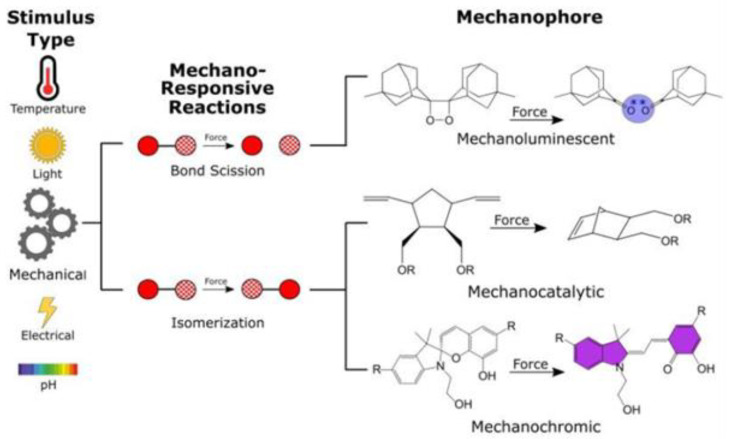
MP definitions and nomenclature: a variety of stimulus types can induce a response in stimuli-responsive materials. Specifically, mechanically responsive materials typically undergo chemical changes, with activation mechanisms shown schematically. Representative reactions for each type of mechanoresponsive molecule are shown. MP-labeled polymers employed as stress sensors can be subjected to a wide variety of loading conditions. Reproduced from Reference [1] with permission from the Royal Society of Chemistry.

To date, some experience has been accumulated in introducing MP into polymers. Among them is the mixed method used in the early stages of work in this area presented by Giardelli et al. [77]. Along with it, the method of inoculation of mechanogenic groups to polymer chains is also used by Paterson et al. [78], Bowser and Craig [63] and Traeger et al. [79]. Figure 5 on the left shows the types of effects, such as temperature, light, mechanical, electrical and chemical effects in the form of changes that can cause a chemical bond break or an isomerization reaction, and the introduced MP will be transformed, while exhibiting mechanoluminescent, mechanocatalytic or mechanochromic properties. The analysis of the results shown in Figure 5 allows us to draw several conclusions. Considering the possibility of introducing into volumetric structures made of polymers subjected to various loads, for example, window panels of aircraft that operate in extreme conditions, which contract, stretch, and vibrate, the authors believe that it is for such conditions that the use of MP is most desirable.

According to Giardelli et al. [77], substances reacting to mechanical stress can be introduced into polymers by extrusion. Typically, the concentrations are in the range of 0.2–2.0 wt.%. At the same time, it is reported that other options for their introduction are possible, for example, through solutions of both components or through solid-phase mixing. Oligo(p-phenilene vinilene, bis(benzoxazolyl)stilbene and perylene bisimides(alkyl-PTCDL) are used as a dye. Another way of introducing MP into polymer media is to graft mechanochromic fragments to polymer chains, which is discussed in detail in [63,78].

New promising methods of introducing MP into polymer objects by 3D printing are reported by Rupp and Binder [80]. Thanks to the use of this method of introducing a mechanophore, its reproducible distribution in the created product is achieved, which is important in mass production. In the above paper, the method of double printing was used, while the polymer and MP were applied separately. Poly(e-caprolactone), polyurethane and alkyl (C11)-based latent copper (1) catalyzed azide/alkine “clic”-reaction of an azide-functionalized fluorescent dye inside a bulk polymer material were used as media. Focus is placed on the printability and postprinting of latent MPs and fluorogenic print components. Chen et al. [81], Calvino et al. [82], Zhang et al. [83] and Yanada et al. [84] showed that choosing the correct MP–polymer combination for a specific application is very important when taking into account the MP activation method and the physico-mechanical properties of the polymer in accordance with the requirements for it.

Currently, the range of MP is constantly expanding, since almost every polymer must have its own MP, and this selection is carried out according to the principle of obtaining the maximum result as shown by O’Neill and Boulatov [85] and Yang et al. [86]. The development of the methodology for the application of MP also continues to expand. In particular, Stratigaki et al. [87] pay attention to the development of methods for loading and measuring the response of a polymer object with MP introduced into its composition. Materials that react to external influences include a number of molecules that react physically or chemically to changes in external conditions, mechanical or electrical influences. In the material reacting to such an impact, a bond break usually occurs or an isomeric rearrangement occurs.

Choosing the proper MP–polymer combination for a specific application is very important [85,86]. The activation of MP depends on the activation method, the mechanical properties and structure of the polymer matrix, as well as the molecular design of the MP itself. PDMS, PMA, PMMA and polyurethane are the most commonly studied polymer matrices for the inclusion of MP. These four polymers are used for commercial purposes because of their availability, wide industrial applications, optical transparency and the relative ease of manufacturing products from them. A wider range of polymers is needed to solve a variety of tasks in which MP can be useful. The choice of a specific MP and its introduction into a specific polymer should be carried out taking into account its activity and the ability of its combination with the polymer matrix [83,87].

Based on the analysis of the literature given in this section and other sources, the following conclusion can be made. It is necessary to continue research in this area and at the same time strive for quantitative calibration of MP activation by correlating the intensity of fluorescence with exposure. It is also necessary to establish for each MP–polymer system the effect of the concentration of the MP on the intensity of its activation. It is important to quantify the effect of MP concentration on the physico-mechanical properties of the initial polymer, which will allow us to find the critical concentration of MP. Another important area of research and their generalization is the creation of a database on the systems “particular polymer-mechanophore” with the inclusion of parameters such as the modulus of elasticity, the beginning of activation of the MP, and quantum yield, which will facilitate the selection of the appropriate MP for a particular polymer, taking into account its operating conditions. The next important task is to develop measuring methods for monitoring the start of the activation of MPs. The solution of these tasks will allow us to move on to solving engineering problems on an industrial scale

### 6.2. New Physical Phenomena Accompanying External Mechanical Effects on Polymers and Relative Substances

The known consequences of mechanical effects on polymers, such as the color change of reaction mixtures in the presence of MP, as it turned out, are only one of the varieties of the results of such physical phenomena. In recent years, the range of observed phenomena has significantly expanded due to the use of original methods and techniques for registering such phenomena. A significant part of such studies was carried out at the Enikolopov Institute of Synthetic Polymeric Materials of the Russian Academy of Sciences, which also led to new laboratory technologies for the synthesis of various substances. In particular, Lyakhov et al. [24] found out that under conditions of rapid compression, nano- and microcomposites based on polystyrene and hybrid nanoscale particles of molecular silicasol of the core–shell type instantly collapse when a certain critical pressure is reached, and this process is accompanied by the emission of electromagnetic and acoustic waves. At the same time, the threshold of critical pressure and the characteristics of the electromagnetic and acoustic waves arising depend on the composition of the composite and the size of the filler particles. The increase in critical pressure with an increase in the concentration of nanoparticles, according to the authors, is associated with the activation of plastic flow in the material during rapid uniaxial compression due to local stresses in the polymer matrix, and they believe that clarification of the mechanism of the observed effects is possible with further research. 

**Figure 6:**
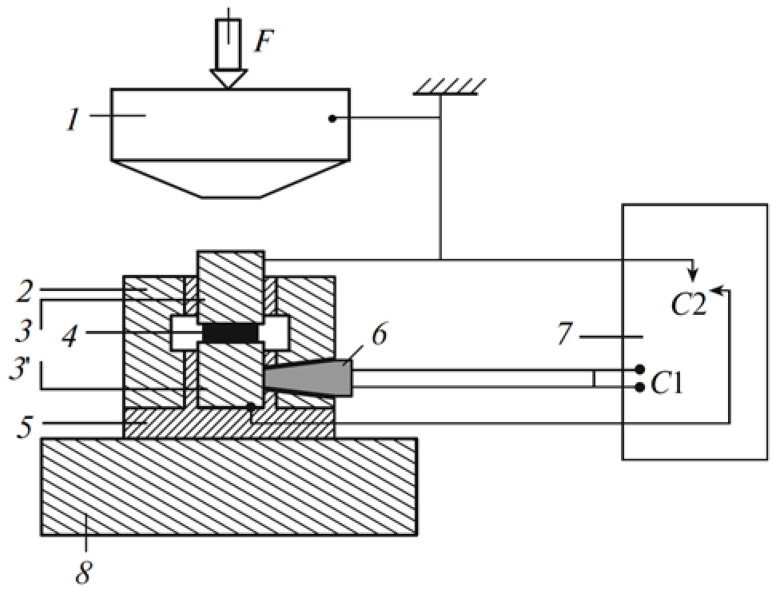
High-pressure cell for the analysis of acoustic and electrical signals during the rheo-logical explosion of polymer samples: (*1*) Bridgman anvil, (*2*) steel casing, (*3*, *3*′) steel waveguides, (*4*) polymer sample, (*5*) insulation, (*6*) ultrasonic transducer with a focusing delay line, (*7*) digital oscilloscope, and (*8*) steel press frame [26].

To carry out these studies, an original high-pressure cell based on Bridgman anvils and a special installation (Figure 6) were used by Aleksandrov et al. [26]. A special feature of this installation is its additional equipment with steel waveguides and an ultrasonic transducer with a focusing delay line, which made it possible to record acoustic and electrical emission signals and determine the frequency spectra of electromagnetic and acoustic waves emitted during an explosion by their changes in time. It should also be noted that the authors were able to observe the transition from a composite with nanoscale particles of silicasol to a composite with predominantly micro-sized particles and that the threshold of critical pressure during an explosion depends on the concentration of the filler and its size in the studied composite. The authors attribute the increase in critical pressure with increasing filler content in the composite to the activation of plastic flow during rapid uniaxial compression of the composite and recognize that this judgment is probabilistic and requires further research.

**Figure 7:**
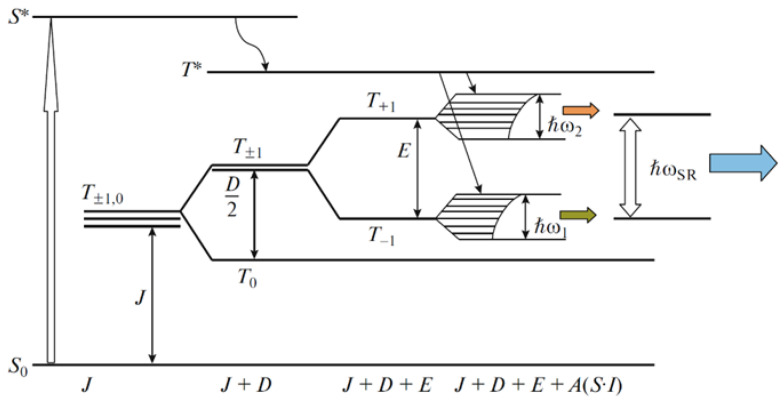
Energy diagram for obtaining the inversion of population of *T*_+1_ and *T*_−1_ levels (the length of horizontal sections is proportional to the population of levels upon splitting of *T*_+1_ and *T*_−1_ by the mechanism of spin–spin interaction) [89].

Subsequent articles by Aleksandrov et al. [88,89,90] report on the established effect of pulsed mechanical activation of radiofrequency super-radiance under the influence of an external rheological explosion. The approach makes it possible to find out how external superradiance affects a sample in which a rheological explosion is excluded [88]. Composites based on polystyrene with additives of Co-(OH) binuclear complexes were studied, Co(OH)_2_-O-Co(OH)_2_ or Mn(OH)_2_-O-Mn(OH)_2_, where OH is a ligand based on 3,6-ditretbutylpyrocatechin. The authors have made some changes to the design of the installation—the cell was isolated from the cage and the punch, between which the test sample was located. The anvils were connected to a digital oscilloscope, which made it possible to register a time-dependent current that occurred during a rapid rheological explosion of polystyrene placed in space in series with the anvils. In this original work, the possibility of implementing the parametric process of radiofrequency superradiation by external mechanical action under high pressure on polymer composites containing paramagnetic particles is shown. Another important result [89] was revealing the regularity of the occurrence of the superradiance regime in the study of the same objects with the Dzyaloshinskii–Moriya effect. The effect is due to electron spin interactions in an electron spin reservoir, so the magnetic field causes magnetic forces. Under the external influence of elastic wave pulses, the electron spin “reservoir” formed by a system of non-collinear spins was shown to be inversely populated. At the same time, it was found that the intensity of superradiance is directly related to the electronic properties and structure formed by two-spin systems with a polymer matrix [90]. The results of the considered works significantly deepened and expanded the information about the physics of mechanochemical effects on polymers. It was shown, in particular, that the intensity of superradiance is related to the electronic properties and structure of two-spin intermediate products–radical pairs and organoelement biradicals detected by ESR [90]. A scheme for initiating a rheological explosion due to the formation of paramagnetic particles with uncorrelated spins was proposed (Figure 7) [89]. Energy pumping of the system upon rheological explosion implements fast transitions of BC–Co and BC–Mn from the singlet state *S* to the excited singlet state S* and then to the excited triplet state T*. The *T*_+1_ and *T*_−1_ levels transform to broad bands and inside these subzones, electron transitions can occur, which are likely to be responsible for low-frequency bands in a range from 0 to 100 MHz. The scheme also explains the superradiance bands at 180.0 and 189.7 MHz. 

A study of the influence of polyethylene chain length on the initiation of rheological explosion showed that because of the explosion, impulses of electric current are formed [91]. Their Fourier images have the form of band spectra, which are described by Lorentz models for a harmonic oscillator with attenuation in the Havriliak-Negami model for dielectric relaxation. The results of the studies by Aleksandrov et al. [90,92] significantly enhance the knowledge about the structural changes undergoing by the studied objects of research due to the rheological explosion, as well as about new electromagnetic and electrical phenomena and due to their analysis. Aleksandrov et al. [92] proposed that a rheological explosion in a polymer sample occurs when a critical concentration of nanoscale pores is reached. The formation of pores, accompanied by the generation of electron–hole pairs (dipoles), is followed by their connection, leading to the formation of pore channels and a change in the charge density in them, and thus an electromagnetic wave and a current pulse are generated.

Another important achievement presented by Aleksandrov et al. [93] was obtained by rheological explosion of a polystyrene composite consisting of a heterospin molecular magnet on Bridgman anvils with rapid pressure release. The composite included the Eu(III)(O)_3_bipyridyl complex at various concentrations. Dynamic spectra were shown to be described in the framework of the Lorentz model for a dynamic oscillator with attenuation.

## 7. Conclusions

The main areas of work in the field of polymer mechanochemistry are developing in the following directions:

(1) Study of the mechanisms and energy of chemical transformations in various mechanical fields;
(2) The use of mechanochemical reactions for the synthesis and modification of polymer materials;
(3) Search for ways to inhibit undesirable mechanochemical side processes that cause a drop in strength, increased wear and premature failure of various parts and structures made of polymer materials during their operation in static and dynamic mechanical fields.

The peculiarity of studies in the field of mechanochemistry is also that they are mostly carried out by the method of searching for positive results. This is primarily due to the fact that it is currently impossible to theoretically predict the expected consequences of such impacts. In this regard, it is important to note the SMFS used as the first step toward developing mechano-sensitive moieties for applications [37].

Along with the directed mechanical actions discussed above, mixing is used in technological practice. Mixing is widely used in various industries, including the production and processing of plastics. In the vast majority of cases, a pure polymer does not have the necessary set of properties and cannot be used for the manufacture of products; therefore, various additives are introduced into the base polymer by mixing (plasticizers, fillers, stabilizers, dyes, pigments, structure-forming agents, other polymers). According to experts, approximately 80% of all manufactured plastics are trained in mixing equipment before processing. Mechanochemical treatment (mechanical milling and alloying) is the mechanical treatment of solid mixtures, as a result of which the plastic deformation of substances occurs, mass transfer is accelerated, the mixing of the components of the mixture at the atomic level is carried out and the chemical interaction of solid reagents is activated [62].

## Figures and Tables

**Table 1:** Comparison of thermal destruction and mechanical destruction of some polymers [69].

Polymer	Volatile Products of Thermal Destruction	Volatile Products of Mechanodestruction	Coincidence of Product Composition
Polymethyl methacrylate (PMMA)	monomer	monomer	yes
Polystyrene (PS)	monomer, oligomers	monomer, dimer	no
Poly-α-methylstyrene (isotactic) (p-α-MS)	monomer	monomer	yes
Polyformaldehyde (PFA)	monomer	monomer, unidentified products	no
Polyethylene (PE)	low molecular weight saturated and unsaturated hydrocarbons	low molecular weight saturated and unsaturated hydrocarbons	?
Polypropylene (PP)	low molecular weight saturated and unsaturated hydrocarbons	low molecular weight saturated and unsaturated hydrocarbons	?
Polyacrylonitrile (PAN)	HC=N, monomer	unidentified products	no
Nitrocellulose (NC)	CO, CO_2_, H_2_O, NO, NO_2_, CH_2_O	CO, CO_2_, H_2_O, CH_2_O	no
Polycapramide (PCA)	H_2_O, CO_2_, NH_3_, hydrocarbons	CO_2_, NH_3_, hydrocarbons	no
Polyepoxide	H_2_, CO, CH_4_, C_2_H_4_, C_2_H_6_, C_8_H_6_, C_8_H_8_	H_2_, CO, CH_4_, C_2_H_6_	no
Polymetaphenylene isophthalamide (PMFIA) (nomex, phenylone); Polyparaphenyleneterefthalamide (PFTA) (kevlar, arenka); Polyparabenzamide; Polyamide benzimidazole (PABI)	NH_3_, H_2_O, CO, CO_2_, benzene	NH_3_, H_2_O, CO, CO_2_	no

**Table 2:** Kinetic parameters of the radical accumulation process during mechanical treatment of polymers at 77 K. *W_0_* is the initiation rate, p* is the maximum concentration of free radicals.

Polymer	*W*_0_·10^−15^, g^−1^ s^−1^	p*·10^−18^, g^−1^
Polyethylene	5	3
	1.7	3
	0.2	5
Polymethyl pentene	2.3	2
Polyethylene oxide	8.3	1
Polystyrene	6.1	5.5
	2.4	6
	0.4	6.5
Polytetrafluoroethylene	0.2	1

## Data Availability

Not applicable.

## Abbreviations

*E*	elastic modulus
*E_kin_*	kinetic energy
*E_vib_*	vibration energy
*U**	amplitude of acoustic waves
*U*	elastic energy
Δ*L*	tensile modulus
σ_p_	tensile strength
AFM	atomic force microscopy
ATRP	atom transfer radical polymerization
ESR	electron spin resonance
MP	mechanophore
MW	molecular weight
Pa	Pascal
PE	polyethylene
PDMS	polydimethylsiloxane
PMA	poly(methyl acrylate)
PMMA	polymethyl methacrylate
SDDAC	structure-deformation diamond anvil cell
SMFS	single-molecule force spectroscopy
SPD	surface plastic deformation
